# A Decision Mixture Model-Based Method for Inshore Ship Detection Using High-Resolution Remote Sensing Images

**DOI:** 10.3390/s17071470

**Published:** 2017-06-22

**Authors:** Fukun Bi, Jing Chen, Yin Zhuang, Mingming Bian, Qingjun Zhang

**Affiliations:** 1 Department of Electronic and Information Engineering, North China University of Technology, Beijing 100144, China; 2 Department of Information and Electronic, Beijing Institute of Technology, Beijing 100081, China; zhuangyin640829@163.com; 3 Beijing Institute of Spacecraft System Engineering, Beijing 100094, China; bianmingming2008@163.com (M.B.); ztzhangqj@163.com (Q.Z.)

**Keywords:** decision mixture model, deformable part models (DPM), decision template, ship detection, remote sensing image

## Abstract

With the rapid development of optical remote sensing satellites, ship detection and identification based on large-scale remote sensing images has become a significant maritime research topic. Compared with traditional ocean-going vessel detection, inshore ship detection has received increasing attention in harbor dynamic surveillance and maritime management. However, because the harbor environment is complex, gray information and texture features between docked ships and their connected dock regions are indistinguishable, most of the popular detection methods are limited by their calculation efficiency and detection accuracy. In this paper, a novel hierarchical method that combines an efficient candidate scanning strategy and an accurate candidate identification mixture model is presented for inshore ship detection in complex harbor areas. First, in the candidate region extraction phase, an omnidirectional intersected two-dimension scanning (OITDS) strategy is designed to rapidly extract candidate regions from the land-water segmented images. In the candidate region identification phase, a decision mixture model (DMM) is proposed to identify real ships from candidate objects. Specifically, to improve the robustness regarding the diversity of ships, a deformable part model (DPM) was employed to train a key part sub-model and a whole ship sub-model. Furthermore, to improve the identification accuracy, a surrounding correlation context sub-model is built. Finally, to increase the accuracy of candidate region identification, these three sub-models are integrated into the proposed DMM. Experiments were performed on numerous large-scale harbor remote sensing images, and the results showed that the proposed method has high detection accuracy and rapid computational efficiency.

## 1. Introduction

High-resolution optical remote sensing images have become an important research topic in many marine applications. Due to their large scale and high efficiency, such images have been extensively used in ship detection, such as in dynamic harbor surveillance, maritime management, ship rescue and smuggling activity monitoring [1,2,3].

In particular, ocean-going vessels and inshore ships are considered typical ship detection scenes. A number of previous studies have focused on ocean-going vessel detection, and they usually showed good performances [4,5,6,7,8,9]. In addition, for inshore ship detection scenes, anchored ships, which are docked in harbor but are not connected to a dock, have similar backgrounds as ocean-going vessel scenes. Given these characteristics, anchored ships were detected effectively in [10,11]. However, in inshore ship scenes, compared with anchored ships, ships berthed at a dock, which are called “docked ships”, are rarely focused on. This is primarily because of the high degree of similarity in gray information and textures between the dock and the docked ship, which are almost connected. These factors make it challenging to accurately detect docked ships from harbor regions.

Therefore, methods have recently been developed to address these intractable problems. Based on detection approach, these detection methods can be divided into three categories. The first category is based on a priori information. Long et al. [12] employs a priori geographic information to rapidly locate harbors. An accurate geographic information system (GIS) contributes to realizing the segmentation between the sea and harbor land and facilitates separating between inshore ships from the harbor. The second category is based on water–land segmentation and contour extraction. These types of methods, which rely on primary image features such as gray information and textures, are proposed in [13,14,15]. An alterable included angle code-based method is proposed by Jiang et al. in [13]. This algorithm for the alterable included angle code is simplified by the evaluation parameter of the broken line in this paper. Xu et al. [14] employs the invariant generalized Hough transform, which could adapt to the translation, scale and rotation transformation of ships, to extract the ship shape. A method based on shape and context information is presented in Liu et al. [15]. Successive shape analyses are proposed in this work to achieve accurate detection of docked ship locations. Recent, model-based methods could be considered to belong to yet a third category. Xu et al. [16] proposes a new detection framework based on the robust invariant generalized Hough transform to detect inshore ships that could adapt to deformations of various ships. The saliency and S-HOG (histogram of oriented gradient) descriptor is presented to detect unsupervised ships by Qi et al. [17]. This method characterizes the gradient symmetry of ship sides to distinguishing between ships and false alarms.

The methods introduced above offer superior detection in certain scenes; however, they have some limitations. Although the methods in the first category are effective, they are limited to specific areas for which accurate GIS information is available and could not be used in unknown areas. During the detection process, water–land segmentation results play an important role in the method of the second category. However, the gray information and textures of inshore areas are complex and are typically influenced by shadows and sunlight illumination. Thus, the segmentation results are unstable. Furthermore, these algorithms are challenging to use to extract continuous contours of docked ships. Ultimately, in practice, it is difficult to effectively distinguish ships from their harbor regions. Recently, many models-based methods that adapt to deformation are presented to detect inshore ships that could be classified as belonging to the third category. However, these models are not applicable to targets that are partly covered by the shadows of higher ship superstructures. The detection accuracy is also influenced by different location angles of docked ships and the complexity of ports. Moreover, these methods would be computationally demanding because detailed scans of the entire image are required to obtain ship-like regions.

**Figure 1:**
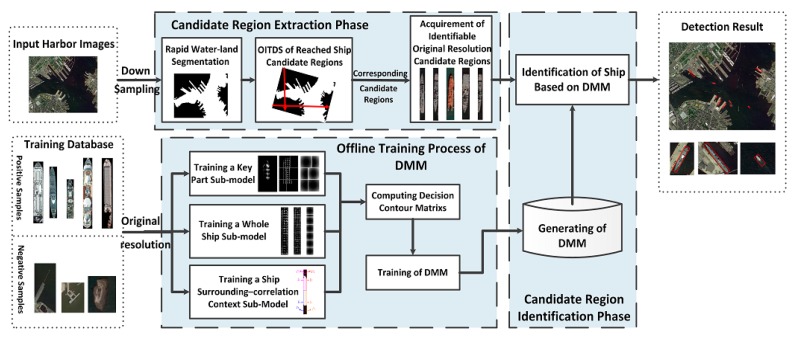
Workflow of the proposed detection algorithm. DMM: decision mixture model.

In this work, a hierarchical framework for inshore ship detection is designed to overcome the aforementioned problems, and Figure 1 shows the algorithm workflow of the proposed method. This framework has two major parts: (1) the candidate region extraction phase, which is conducted at a rapid speed and presents fewer missed errors, and (2) the candidate region identification phase, which has high precision and a lower false alarm rate value. In the candidate region extraction phase (part (1)), a rapid screening method is presented to extract candidate regions of inshore ships. Specifically, the candidate regions of anchored ships in water areas could be easily extracted based on fusing the gray information and texture information. Furthermore, an omnidirectional intersected two-dimension scanning (OITDS) method is presented to quickly extract candidate regions of docked ships from coastal areas in large scenes without GIS information. In the candidate region identification phase (part (2)), we propose a decision mixture model (DMM) to effectively differentiate ships from candidate regions. Due to the robustness to deformation, we use a deformable part model (DPM) as proposed by Felzenszwalb et al. [18,19] to construct the key part sub-model and whole ship sub-model of a single ship. Then, a ship surrounding correlation context sub-model is designed. Finally, the scores of these three sub-models in each candidate region are fused into a decision template (DT), as proposed in [20], to effectively discriminate between ships and false alarms. Previous work could not extract valid candidates caused by discontinuous contours; however, the proposed DMM resolves these issues. The DMM uses a small number of sub-models to describe a ship and ensure the robustness of identification to account for different ship appearances. To improve the detection accuracy, the feature information from the three sub-models is used in the DMM to identify ships. Therefore, the DMM could not only deal with typical scenes of docked ships with different location angles but could also adapt to non-optimal scenes, such as docked ships partially obscured by shadows from a higher ship’s superstructure and scenes in which the gray features of a docked ship are extremely similar to the harbor area, etc.

## 2. Extraction of Candidate Regions

Distinct contrasts occur between anchored ships and their surrounding water; therefore, extracting candidate regions of anchored ships is simple. However, the similar features between docked ships and docks increase the difficulty of extracting candidate regions of docked ships. Docked ships always present a protruding characteristic in relatively smooth areas along the coast; therefore, when searching for this characteristic, the rapid and low-error method OITDS is designed to extract candidate regions of docked ships. Additional details are presented below.

### 2.1. Rapid Water–Land Segmentation

Water–land segmentation can help extract the candidate regions of two types of inshore ships. To improve efficiency, down-sampling is used to input large-scale images. After sampling, the gray feature distribution and texture neighborhood variance distribution from the sampling images are calculated. Based on the peak characteristics of these two features, an adaptive segmentation threshold could be obtained to preliminarily extract the harbor water areas. According to the ship-like shape and size characteristics of most ships, false candidate regions that are obviously incorrect can be deleted. Finally, water segmentation binary images are obtained. A more complete description can be found in [10]. Suppose that F(x,y) is the location feature of a pixel I(x,y) in these binary images. The feature of the pixel located in the land area is labeled F(x,y)=1, and the feature of the pixel located in the water area is labeled F(x,y)=0.

After water–land segmentation, the obvious candidate regions of anchored ships can be obtained from the binary images. Due to the similar features and the close connection between the ship and dock, a docked ship is often misjudged as a land area. Therefore, further separation is necessary to distinguish docked ships from the obtained land area, as presented in the following section.

### 2.2. OITDS of Docked Ship Candidate Regions

A docked ship presents a protruding characteristic in a smooth area along the coast. Considering this characteristic, intersected two-dimension scanning (ITDS) is used to extract the candidate from the binary images of water–land segmentation. First, the intersected scanning of the vertical and horizontal directions finds the coastline (sea–land alternating pixels) which has two adjacent pixels F(x,y) change from “0” to “1” or from “1” to “0” in the binary images. Then, the F(x,y) locations of these pixels that satisfy the coastline location are changed to “2”. When the coastline is ensured, bulges surrounded by coastline could be found because of their protruding characteristics. Thus, in each vertical and horizontal direction, the positions between each pair of coastline pixels may be a part of the bulging areas. If these location features are labeled “1”, these positions are parts of the bulge in the coast. Therefore, these pixels are instead labeled as “3” and considered as candidates of a docked ship. Eventually, pixels labeled F(x,y)=3 are accumulated and the suspected bulge areas that connect smooth coastal area and water area are obtained. Thus, these pixels labeled as “3” represent docked ship candidates.

**Figure 2:**
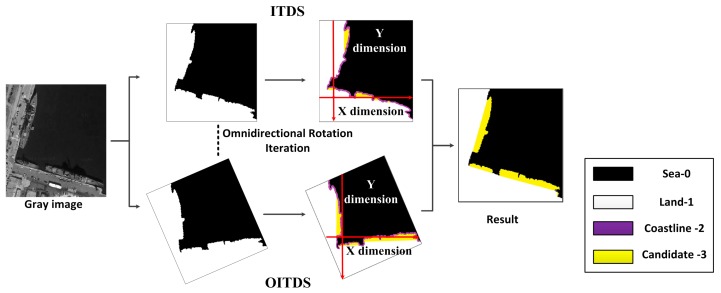
Omnidirectional intersected two-dimension scanning (OITDS) process.

Due to the various locations of docked ships and their different location angles, ITDS cannot easily capture all the docked ships. To overcome this problem, an omnidirectional intersected two-dimension scanning (OITDS) technique is designed that is based on the omnidirectional rotation iteration of ITDS, as shown in Figure 2. This method rotates the binary images to scan as many suspected protruding bulges as possible in different directions. Furthermore, the results of all the directions are synthesized to obtain the vast majority of suspected protruding bulges in the coastal area. In addition, based on the geometric appearance information of ships, such as their length, width and aspect ratios, these features are used to roughly discriminate the scanned results. After primary discrimination, the minimum enclosing rectangles of these bulges are set as the suspected candidate regions (SCRs).

### 2.3. Acquirement of Identifiable Candidate Region

**Figure 3:**
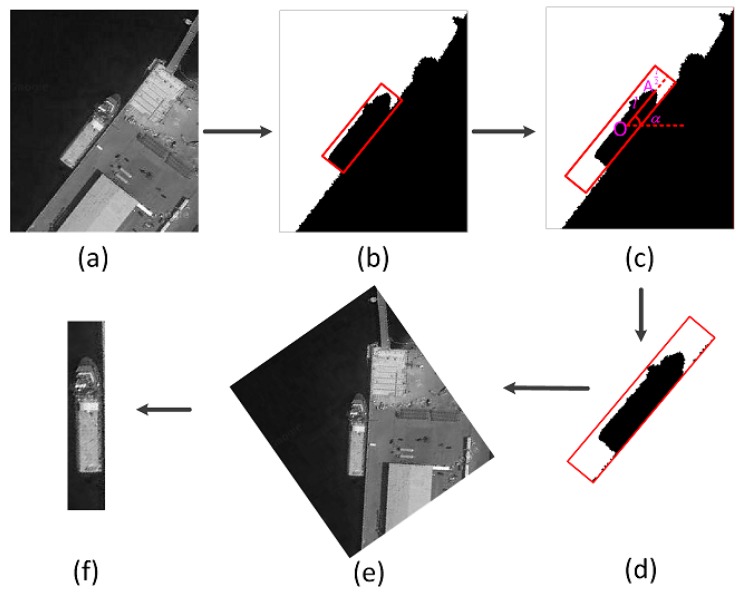
Process of acquiring identifiable candidate regions. (**a**) Part of the input image; (**b**) Suspected candidate regions (SCR) in the binary image; (**c**) Expansion process of the SCR; (**d**) New rectangle after expansion; (**e**) Part of the input image rotated by α; (**f**) New candidate region of the bulge.

To facilitate the subsequent candidate region identification processing, particularly the analysis and identification of ship surrounding correlation context, we need to acquire the identifiable candidate regions. The geometric center point O of the suspected protruding bulge in each SCR is calculated, and point A, which is the maximum distance from the center point O to the bulge, is identified. The length of the line between A and O is l, and the angle α between $\vec{OA}$ and the horizontal direction is calculated. Along the long axis to a point l/2 from both ends of the SCR, a new rectangle is structured as shown in Figure 3. The original resolution gray scale image can be rotated by the angle α to determine the upright area of the new rectangle in the gray image and construct the new candidate region for the bulge. This type of candidate region would be distinguished during the candidate region identification phase. Note that the bulge in the candidate region is in a vertical state at this time.

## 3. Candidate Region Identification Based on Decision Mixture Model

Based on the candidate regions, a DMM strategy is proposed to distinguish between ships and false alarms in this section. First, a DPM is employed to build the key part sub-model and the whole ship sub-model of a ship. Second, according to the context features of the ship bow and stern, a local context for docked ship discrimination is designed by the surrounding correlation sub-model. Finally, the DMM was used to fuse the scores of the three sub-models to achieve reliable ship detection in the candidate regions.

### 3.1. Decision Sub-Models

#### 3.1.1. Key Part and Whole Ship Sub-Models

Although various ships are observed in the inshore area, the bow and hull of single ships have relatively constant structural features. Therefore, these structural features could be described by the deformable models. Specifically, the bow is recognized as a typical ship feature and considered the key part of a ship for identification. The excellent adaptability of object deformation has been presented in other works [21,22,23]; therefore, the DPM introduced in [18,19] is employed to train a key part sub-model and a whole ship sub-model for the proposed DMM framework.

First, histogram of oriented gradient (HOG) feature maps for each input image are constructed by calculating 8 × 8 adjacent pixel blocks according to Dalal et al. [24]. A pyramid map of features at the λth level for each input image, H, is constructed at a multi-scale resolution. In this work, both the key part feature sub-model and the whole ship feature sub-model can be defined by the (n+2) tuple as follows:

(1) $$M=(F_{0},(F_{1},v_{1},d_{1}),(F_{2},v_{2},d_{2}),\ldots ,(F_{n},v_{n},d_{n}),b)\;i=1,2,\cdots ,n$$

where $F_{0}$ is a root model (root filter), $F_{i},v_{i},d_{i}$ is a series of part models, n is the number of part models, and b is a necessary real-valued bias term. In each part model, $F_{i}$ is a part filter and $v_{i}$ is a two-dimensional vector, which indicates the relative position between the anchor position of part i and the root position. In addition, $d_{i}$ is a four-dimensional vector that specifies the coefficient of a quadratic function defining a deformation cost for each possible position of part i relative to $v_{i}$. Both the root filter and the part filter are sized w×h and calculated in H. The part filters are HOG [24] detectors reshaped as liner filters. The root filter is designed to determine the approximate position of the suspected object, and the part filter is designed to determine the precise information of the suspected parts. Therefore, the root filter is calculated at the coarse level, and the part filters are calculated at the finer levels of the pyramid. In addition, the bias term is the deformation cost that realizes the deformation of the filters.

The object hypothesis presents the location of the root filter and the part filters in H, $z=(p_{0},\ldots p_{n})$, where $p_{i}$ defines the level and position of the filter in H. The score of the object hypothesis is given by the score of each filter at their locations minus a deformation cost that depends on the relative position of each part to the root and with the addition of bias,

(2) $$score(p_{0},\ldots ,p_{n})=\sum_{i=0}^{n}{F^{\prime }}_{i}\cdot \phi (H,p_{i})-\sum_{i=1}^{n}d_{i}\cdot \phi_{d}(dx_{i},dy_{i})+b$$

where ${F^{\prime }}_{i}\cdot \phi (H,p_{i})$ is the score of $F_{i}$ in H with the upper left corner in $p_{i}$, ${F^{\prime }}_{i}$ is the concatenation of the rows of $F_{i}$, $\left(dx_{i},dy_{i})=(x_{i},y_{i})-(2(x_{0},y_{0})+v_{i}\right)$ gives the displacement of the ith part filter relative to the anchor location, and $\phi_{d}(dx_{i},dy_{i})=(dx,dy,dx^{2},dy^{2})$ is the ith part filter deformation feature. A more complete description can be found in [18,19].

Both the key part sub-model and the whole ship sub-model are constructed by the method introduced above. Specifically, these two sub-models could structure the complementarity between the whole and key parts of a ship in the identification section. In other words, the sub-models are conducive to overcoming certain non-ideal conditions in which a ship is covered by shadows from the higher superstructure. In addition, the sub-models are also considered to be adaptive to the intra-class deformations of various ships.

#### 3.1.2. Ship Surrounding Correlation Context Sub-Model

Due to the different docking locations and the variety of docking angles of docked ships, identifying a uniform context representation in a scene level for a docked ship is difficult. However, in the majority of docking situations, the surrounding areas of both the bow and stern have obvious context features. Compared with protruding artificial constructions located in coastal areas, the areas surroundings both the bow and stern of a ship are usually water. Furthermore, the gray information and texture features of these two local water regions are similar to those of the water in the inshore area. Thus, a ship surrounding correlation context sub-model is built to describe this feature.

**Figure 4:**
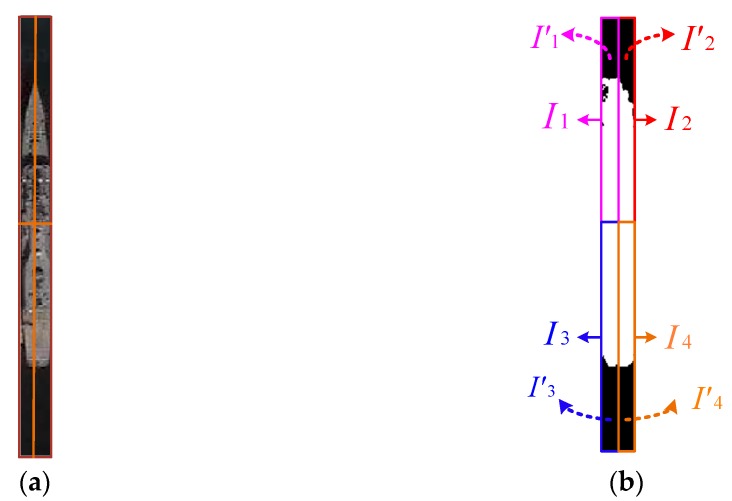
Schematic of ship surrounding correlation context sub-model blocks. (**a**) Candidate region captured from Section 2.3; (**b**) Blocks and water area of the candidate region.

Vertical candidate regions captured from Section 2.3 are translated into binary images based on the adaptive segmentation method introduced in Section 2.1. Depending on the vertical and horizontal symmetry axes, each binary image of the candidate region is evenly divided into a number of blocks ($I_{1},I_{2},\ldots ,I_{N}$) in this work, we set N=4. In each block, the area that can be considered water is labeled $I^{\prime }k$ as shown in Figure 4. Subsequently, features could be calculated from the corresponding position of $I^{\prime }k$ in the original candidate regions. Let $F_{1}({I^{\prime }}_{k}),F_{2}({I^{\prime }}_{k}),\ldots ,F_{M}({I^{\prime }}_{k})$ represent the set of features, where M is the number of features. The gray mean and standard deviation are expressed as $F_{1}({I^{\prime }}_{k})$ and $F_{2}({I^{\prime }}_{k})$, respectively. The contrast feature and correlation feature are extracted from the gray level co-occurrence matrix and could be represented by $F_{3}({I^{\prime }}_{k})$ and $F_{4}({I^{\prime }}_{k})$, respectively. Consequently, the neighborhood descriptions are four-dimensional, M=4. Two of the dimensions are gray descriptors and the other two dimensions are texture descriptors.

In addition, it is important to ensure that the surrounding areas of the bow and stern have similar characteristics, which are defined as follows:

(3) $${\bar{R}}_{k}(i)=\frac{1}{N-1}\sum_{j\neq k}F_{i}\left({I^{\prime }}_{j}\right)$$

(4) $$DF(i,k)=\left|F_{i}\left({I^{\prime }}_{k}\right)-{\bar{R}}_{k}(i)\right|/{\bar{R}}_{k}(i)$$

where ${\bar{R}}_{k}(i)$ is the average of the features $F_{i}$ that are extracted from the water except for ${I^{\prime }}_{k}$ and DF(i,k) is the diversity between ${I^{\prime }}_{k}$ and the other water areas with the same feature $F_{i}$. Finally, a function is used to judge the similarity of each water area in a block as follows:

(5) $$SDF\;=\frac{\sum_{i}\sum_{k}DF(i,k)}{MN}$$

where SDF is the similarity value of each ${I^{\prime }}_{k}$, M is the number of descriptions and N is the number of blocks. This type of similarity function could denote the correlation context of the majority of docked ships. However, harbor wharfs, quay cranes and other facilities, that could easily produce disturbances during detection, would not have this type of similarity in coastal areas.

### 3.2. DMM Based on Decision Template

For each candidate region, by normalizing the outputs of the above three sub-models, the DT ensemble method in [20] is employed to achieve reliable identification of suspected ship candidates as described below.

#### 3.2.1. Training of DMM

First, for each candidate region, identification scores are calculated using the above three sub-models. Different dynamic ranges are observed for each sub-model output; therefore, the outputs are normalized to [0,1].

The three sub-models introduced above are used as decision factors $\left\{D_{1},D_{2},D_{3}\right\}$ in this section. Specifically, the key part sub-model is first the factor $D_{1}$, the whole ship sub-model is $D_{2}$ and the context sub-model is $D_{3}$. There are two classes of suspected objects in the candidate region. One is the ship object presented by $w_{1}$ and the other is the false alarm presented by $w_{2}$. Let $Z_{j}=\{z_{j},1,z_{j},2,\ldots ,z_{j},m,\ldots ,z_{j},N\}$ denote a training database of class $w_{j}$, where $z_{j,m}$ is a database sample and N is the number of training samples. The decision contour matrix is employed to denote the decision habit of $D_{i}$ to $w_{j}$, which is defined as follows:

(6) $$DP(z_{j,m})=\left[\begin{array}{ll} d_{1,1} & d_{1,j}\\ \cdots & \cdots \\ d_{i,1} & d_{i,j} \end{array}\right]\;(i=1,2,3;j=1,2)$$

where $DP(z_{j,m})$ is the decision contour matrix and $d_{i,j}$ is the detection result of decision factor $D_{i}$ to the $w_{j}$. When the result is closer to $w_{j}$, the numerical value of $d_{i,j}$ is closer to 1; otherwise, it is closer to 0.

Afterward, the decision template $DT_{j}$ is used to represent the class of the suspected ship object $w_{j}$, and it is defined as follows:

(7) $$DT_{j}=\frac{1}{N_{j}}\sum_{z_{m}\in w_{j}\in Z}DP(z_{j,m})\;j=1,2$$

The average value of $DP(z_{j,m})$ presents the general decision habit of the decision factors to $w_{j}$ with the corresponding training data. According to this formula, the DMM of a ship object is represented as $DT_{1}$, while a false alarm is represented as $DT_{2}$.

#### 3.2.2. Ship Identification Based on DMM

The DMM is used to confirm ship objects in the candidate regions $z_{ROI}$. By calculating the decision contour matrix of the candidate region $DP(z_{ROI})$, the similarity between the candidate and $DT_{j}$ could be calculated as follows:

(8) $$\mu_{j}(z_{ROI})=\sqrt{\sum_{x_{i}=1}^{2}\sum_{y_{j}=1}^{3}{\left[DPZ_{ROI}(x_{i},y_{j})-DT_{j}(x_{i},y_{j})\right]}^{2}}$$

where $\mu_{j}(z_{ROI})$ is the similarity value between $DP(z_{ROI})$ and $DT_{j}$. According to the different categories $w_{j}$, two types of Euclidean metrics {$\mu_{1}(z_{ROI})$, $\mu_{2}(z_{ROI})$} are required.

The minimum value of the metrics μk(zROI) indicates that $w_{k}$ is the class of the suspected object in $z_{ROI}$. Furthermore, a ship object could be confirmed in $z_{ROI}$. When there is a ship in $z_{ROI}$, the minimum bounding rectangle of the ship can be obtained, and that rectangle can be shown in the remote sensing image at the original resolution.

## 4. Results and Evaluation

A number of experiments are designed and evaluation methods are presented in this section. Eighty harbor remote sensing images were gathered from Google Earth. These images were approximately 6000 × 8000 pixels in size and had resolution from 1 m to 1.7 m per pixel. A large number of ships with different shapes and location angles could be observed in these images. For sub-models training and DMM training, ship candidate region patches were captured from 50 images to build the positive sample training database. Note that each of these image patches contains only one ship, and these patches contained the vast majority of ship shapes. In addition, image patches of interference false alarms were found, such as ports, convex coasts, islands, etc., at sizes ranging from 400 × 100 pixels to 600 × 200 pixels. These patches were used to compile a negative sample training database. Furthermore, the remaining 30 images were used to build the testing database. This database contained a total of 350 ships located at different angles and multiple shapes in inshore areas. Herein, certain performance metrics are defined as follows:

(9) $$Recall=\frac{\mathrm{number}\;\mathrm{of}\;\mathrm{real}\;\mathrm{detected}\;\mathrm{ships}}{\mathrm{total}\;\mathrm{number}\;\mathrm{of}\;\mathrm{real}\;\mathrm{ships}}$$

(10) $$Precision=\frac{\mathrm{number}\;\mathrm{of}\;\mathrm{real}\;\mathrm{detected}\;\mathrm{ships}}{\mathrm{total}\;\mathrm{number}\;\mathrm{of}\;\mathrm{detected}\;\mathrm{results}\;}$$

Please note that the total number of real ships is the sum number of both anchored ships and docked ships.

### 4.1. Key Parameter Analysis

**Figure 5:**
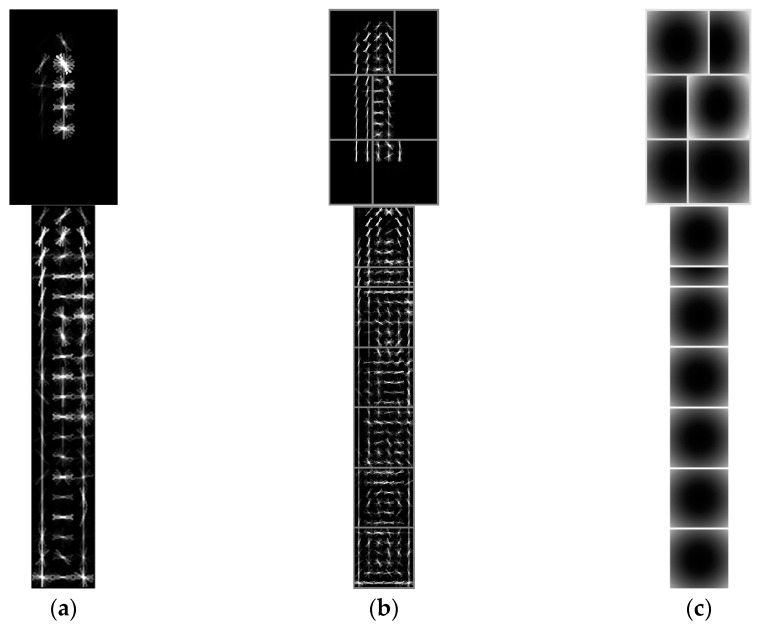
Key part sub-model and whole ship sub-model. (**a**) Root model; (**b**) Part model; (**c**) Spatial location models.

In the proposed detection method, the key part sub-model and the whole hull sub-model play critical roles in the DMM. In Figure 5, the first row shows the key part sub-model and the second row shows the whole ship sub-model. 

**Figure 6:**
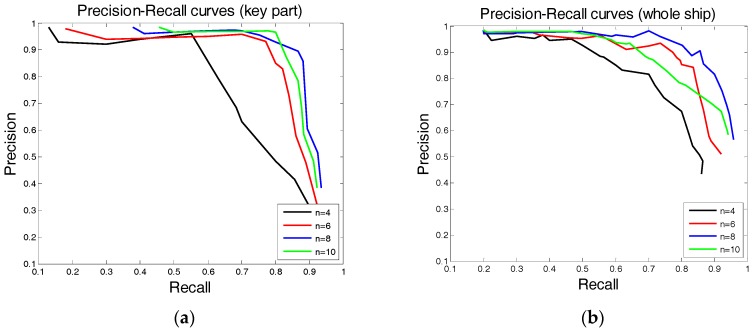
Precision-recall curves as parameter n varies: (**a**) key part sub-model detection results when n=4,6,8,10 and (**b**) whole ship sub-model detection results when n=4,6,8,10.

There is one key parameter n, which represents the number of part filters in the sub-model. The ability of the model to describe the target is closely related to this parameter. In addition, this parameter influences the capacity of the model to resist object deformation. Thus, certain experiments for this key parameter were structured to assess how the detection performance was affected by the value of n. We tested the key part sub-model and whole ship sub-model respectively in the test database we built and there are 30 images including 350 real ships that were involved in the test. As presented in Figure 6, the recall and precision curves of the detection results have a certain trade-off relationship.

When large recall is required, the precision value must be reduced and vice versa. To obtain the best detection result, a stable detection effect should be selected, and both the recall and precision should be at a high level. Values of n=4,6,8,10 were tested; the detection results are shown in Figure 6. And a detection-sensitive threshold is set as a tuning parameter of this experiment. This threshold was used to filter scores of object detection. Specifically, the best detection effect is obtained in both the key part sub-model and the whole ship sub-model when n=8. Consequently, n=8 in this work.

### 4.2. Detection Result Analysis

**Figure 7:**
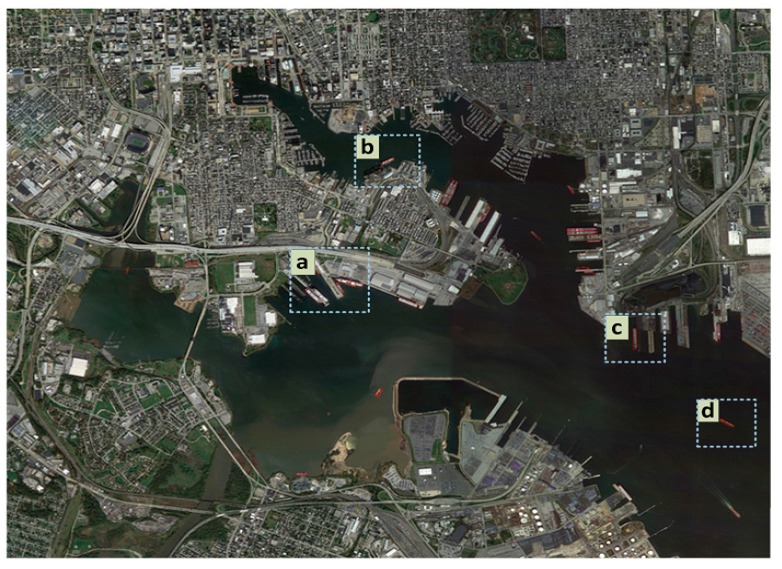

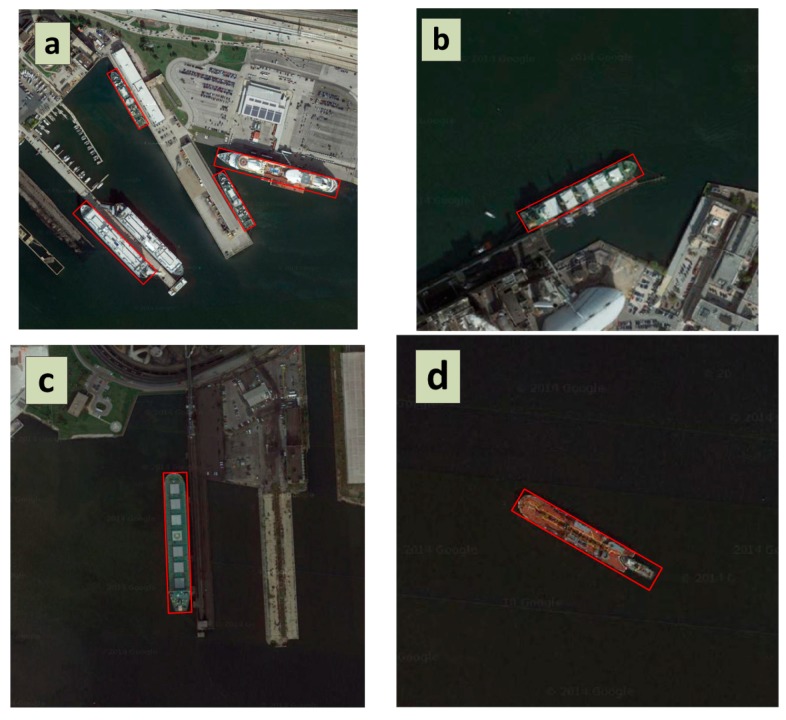
Detection results of the proposed method.

A typical detection result of the proposed algorithm in a large-scale remote sensing harbor image is presented in Figure 7. In addition, selected typical local results from the large-scale harbor image are taken as examples. The proposed method could successfully address diverse harbor locations, complex illumination conditions and different ship location angles. Furthermore, this approach also has a better adaptability to shadow interference that occurs on water and ship decks. Therefore, this method has good robustness for complex scenes. However, it is unable to detect a few ships because of more specific interference factors (e.g., dock facilities) connected to the bow and stern at the same time, as shown in Figure 7a. These specific interference factors may lead to a low score in the ship surrounding correlation context sub-model. Thus, this scene would affect the final decision.

To demonstrate the advantages of this method, it was compared with other typically used methods as applied in [15,16] as well as a basic DPM method. The comparison results are shown in Table 1. To ensure the fairness of the experiment, the same database was used for all the methods, and the parameters of the contrastive algorithms were adjusted to the optimal state.

As shown in Table 1, the basic DPM, the method applied in [15] and the method applied in [16] could not achieve better detection results than the proposed method. Method [15] achieves lower recall in these experiments; it is designed to analyze binary images obtained after water–land segmentation. These segmentation results are influenced by the shooting and illumination angles. In addition, it requires a large number of parameters in the analysis stage. Specifically, method [16] had lower precision because it relies on contour information, particularly bow contour information. Moreover, the structural characteristics of whole hulls are considered less. In contrast, the proposed method focuses on complementary both bow and hull features of a ship. In contrast, the proposed method is based on gray images to achieve object detection and obtain a decision template via automatic learning. Obviously, the basic DPM method, which is based on only a one hull sub-model, has the lowest recall and precision because it lacks consideration of bow features and context features.

The proposed method also has fast processing speed. These three methods always focus on image patches and must meticulously scan all regions in the image patches. When dealing with large-scale harbor remote sensing images, their calculation process is intensive. However, the proposed method works by structuring a hierarchical detection framework; therefore, extracting candidate regions is a coarse-to-fine process. The small-sized images of local candidates are roughly captured from large-scale images. Thus, the precise detection function could gradually focus on the target area and it does not require globally detailed computing processes. This algorithm structure effectively reduces the calculations required to perform detection.

## 5. Conclusions

In this paper, a hierarchical framework for inshore ship detection in large-scale harbor remote sensing images is presented. To improve the efficiency, a novel scanning method, OITDS, is proposed for candidate region extraction. This method ensures that candidate regions can be extracted from large-scale images at high speed and with fewer errors. To enhance the robustness of detection in complex scenes, a DMM strategy is presented to confirm the captured candidate regions. The whole ship features, the key part features and the context features of ships are fused into the DMM strategy. This comprehensive decision method fully considers the adaptability of target deformation and increases the robustness on unsatisfactory scenes. Experiments on large-scale harbor remote sensing images verify that the proposed method is effective and robust when applied to unsatisfactory scenes. Compared with typical methods, the proposed method also achieves better detection results. This method aims at rapidly getting the berthing situation of inshore ship from interested port by using a wide range of remote sensing images. Especially, some situations that traditional information acquisition methods could not deal with (such as non-cooperation) would suit our method. In the future, based on the proposed detection method, we plan to identify and classify inshore ships. Moreover, additional data for different types of ships in inshore areas will be analyzed. The proposed method will benefit the integrated harbor management and support shipping management and other activities.

## Figures and Tables

**Table 1:** Detection Results of Different Methods.

Method	Recall	Precision
Ship detection-based method [15]	73.5	81.3
Ship detection-based method [16]	80.4	71.9
Basic DPM method	64.2	58.1
Proposed ship detection method	92.4	85.6
